# The Effects of Yacon Consumption on Body Weight and C-reactive Protein: A Systematic Review and Meta-analysis of Randomized Controlled Trials

**DOI:** 10.1016/j.curtheres.2025.100817

**Published:** 2025-12-17

**Authors:** Fariborz Porbafrani, Mohammad Samadi, Karim Parastouei

**Affiliations:** 1 Department of Nutrition and Food Hygiene, Faculty of Health, Baqiyatallah University of Medical Sciences, Tehran, Iran; 2 Exercise Physiology Research Center, Life Style Institute, Baqiyatallah University of Medical sciences, Tehran, Iran

**Keywords:** body weight, C-reactive protein, meta-analysis, randomized controlled trial, yacon

## Abstract

**Background:**

The rising global challenges of obesity and chronic inflammation contribute to an increased prevalence of metabolic disorders and cardiovascular diseases.

**Objective:**

The goal of the meta-analysis of randomized controlled trials (RCTs) was to assess the impact of yacon consumption on body weight and levels of C-reactive protein (CRP).

**Methods:**

Online databases such as PubMed/Medline, Scopus, Web of Science, and Google Scholar were searched until June 2024 to collect RCTs that investigated the impact of yacon consumption on body weight and CRP. Meta-analyses were conducted using a random-effects model, and the *I*^2^ index was employed to evaluate the heterogeneity among the RCTs.

**Results:**

Seven studies with a total of 246 participants were included in the study. The pooled effect size indicated that yacon intake was associated with weight reduction (weighted mean difference [WMD], −8.22 kg; 95% CI, −16.01 to −0.43; *P* = .039). In contrast, yacon consumption showed no significant effects on body mass index (BMI) (WMD, −1.48 kg/m^2^; 95% CI, −4.11 to 1.14; *P* = .268), waist circumference (WC) (WMD, −3.73 cm; 95% CI, −10.18 to 2.72; *P* = .257), and CRP (WMD, −0.07 mg/L; 95% CI, −1.96 to 1.81; *P* = .939). Subgroup analyses demonstrated that yacon consumption led to significant weight reductions in individuals aged 40 years and older, as well as in women and those who consumed yacon for more than 8 weeks. Additionally, yacon consumption was associated with reductions in BMI and WC in women and individuals aged >40 years. However, no statistically significant differences were observed for other factors.

**Conclusions:**

The findings suggest that yacon consumption may contribute to weight reduction in adults, though effects on BMI, WC, and CRP remain uncertain.

## Introduction

Overweight and obesity have emerged as significant global health issues due to their strong association with a high prevalence of various chronic diseases, including type 2 diabetes, hypertension, coronary heart disease, and other noncommunicable conditions.^1^^,^^2^ The rising global challenges of obesity and chronic inflammation contribute to an increased prevalence of metabolic disorders and cardiovascular diseases.^3^ In 2022, approximately 2.5 billion adults were overweight, with 890 million living with obesity.^4^ With such a significant portion of the population affected, there is a critical need for effective dietary interventions. Nutritional strategies offer alternatives to pharmaceutical methods for reducing hyperglycemia and body weight.^5^ Identifying novel foods that promote satiety or reduce energy density can be valuable tools in managing obesity and its related comorbidities.^5^^,^^6^

Yacon is a perennial plant from the Andes, valued for its sweet roots with high water content. Both its roots and leaves are traditionally used to treat hyperglycemia in diabetic patients.^7^^,^^8^ Studies have shown that consumption of fructans strengthens the immune system.^9^

Long-term use of yacon syrup for 30 days decreased levels of cholesterol and total body fat.^10^ By contrast, yacon flour consumption for 6 weeks increased body weight, waist circumference (WC), waist to height ratio, and reduced body fat in adults with excess body weight.^10^ However, consumption of yacon flour for 6 weeks increased the plasma antioxidant capacity, but no significant change was seen in levels of plasma C-reactive protein (CRP).^11^

Another randomized controlled trial (RCT) indicated that freeze-dried powdered yacon for 9 weeks can reduce high sensitivity-CRP levels.^9^ Previous reviews on the health effects of yacon have indicated that its consumption may enhance glycemic control and improve lipid profiles.^12^ However, these studies did not include a meta-analysis. In contrast, our work involves conducting a systematic review to comprehensively evaluate and synthesize the existing evidence.

Although several trials have shown beneficial effects of yacon utilization on the improvement of anthropometric and inflammatory indices,^9^^,^^10^^,^^13^ a number of studies have shown contradictory results in this regard.^9^^,^^11^

Due to conflicting evidence from existing trials regarding the protective effects of yacon on inflammatory markers and its beneficial impact on body weight, as well as the absence of a comprehensive meta-analysis, this study was designed to investigate the effects of yacon consumption specifically on body weight and CRP levels.

## Methods

We conducted and provided this meta-analysis in accordance with the Preferred Reporting Items for Systematic Reviews and Meta-Analyses (Supplementary Materials).^14^

### Search strategy

The studies included in this systematic review and meta-analysis of RCTs, published up to June 4, 2024, were identified through a comprehensive literature search in the online databases of PubMed, Institute for Scientific Information (ISI) draws on Web of Science, and Scopus. Throughout the search strategy, the following keywords (MeSH and non-MeSH terms) were used: (Yacon[tiab] OR "*Smallanthus sonchifolius*"[tiab]) AND (intervention[tiab] OR randomized[tiab] OR RCT[tiab] OR Randomly[tiab] OR random[tiab] OR Placebo[tiab] OR trial[tiab] OR randomised[tiab] OR trials[tiab] OR Cross-Over[tiab] OR "Placebos"[Mesh]) to include all RCT studies (Supplementary Materials).

Additionally, searches were conducted in clinical trials registries and on Google Scholar, which yielded the top 30-page results, thereby ensuring thorough coverage of relevant research. In order to avoid overlooking any publications, pertinent articles were also manually searched through the reference lists of the research that was found. The screening process includes all available studies, with no restrictions on language or publication date.

### Study selection

The Population, Intervention, Comparator, Outcomes, Study design criteria were used in the following manner to achieve this goal: (1) Participants: adults over the age of 18 years, (2) Intervention: due to their identical fructooligosaccharide (FOS) composition and modes of action, several yacon forms (syrup, flour, or powder) were mixed together, (3) Comparator: placebo or control groups not supplementing with yacon, (4) Outcomes: studies presenting at least one of the following outcomes: body weight, body mass index (BMI), WC, or CRP, (5) Study design: RCT. The included studies were those that provided effect sizes as mean ± SD or mean ± SEM. Studies with incomplete data (missing baseline, outcome, or change data), observational, editorial, letters to the editor, ecological, review, or comment papers were excluded. Furthermore, studies that did not include a control group, random allocation, or animal model, as well as those involving children or adolescents, were not included. Theses, conference abstracts, and unpublished data are examples of gray literature that were omitted due to a lack of peer review and inadequate methodological reporting.

All of the abstract studies and citations that were found were imported into the EndNote program for review. After removing duplicate citations, the remaining studies were imported into EndNote software for further evaluation. Unpublished research was not included. The studies were assessed separately by 2 researchers in relation to the eligibility requirements. The 2 reviewers (F.P., M.S.) discussed and resolved any disputes that occurred during the evaluation. The study supervisor was responsible for resolving any disputes between the reviewers (K.P.). A high level of agreement was demonstrated by the Cohen’s κ of 0.78 between the assessments made by the 2 researchers (F.P., M.S.).

### Data extraction

Both the reviewers (F.P., M.S.) separately extracted the data and cross-checked. When reviewers disagreed, the principal investigator (K.P.) consulted. To assess the effect of yacon consumption on body weight and CRP, before and after the intervention, we gathered and presented the mean and SD data for these variables for both the intervention and control groups. Each group’s mean and SD changes in weight, BMI, WC, and CRP were determined. Furthermore, data on the first author’s name, publication year, study design, location, health status, sample size, duration (weeks), sex, mean age (years), baseline BMI (kg/m^2^), intervention group, comparator group, and outcome were obtained.

### Risk of bias assessment

The quality of each study was independently evaluated by 2 researchers (F.P., M.S.) using the Cochrane Risk of Bias Tool version 2.0 (RoB 2) for interventional studies.^15^ In cases of disagreement, the reviewers collaborated to reach consensus, and if necessary, consulted a third investigator (K.P.) for resolution. The RoB 2 tool assesses risk of bias across 5 domains: bias arising from the randomization process (D1), bias due to deviations from intended interventions (D2), bias related to missing outcome data (D3), bias in outcome measurement (D4), and bias in the selection of reported results (D5).^15^ Each domain was assessed and classified as indicating a low risk of bias, some concerns, or a high risk of bias based on responses to specific signaling questions within the RoB 2 tool framework. The overall risk of bias for each study was assigned based on the most severe risk level observed in any domain, with studies classified as high risk if at least one domain was rated high risk, and as having some concerns if any domain raised uncertainty issues.

### Grading quality of evidence

The certainty of evidence for each outcome was evaluated according to the Grading of Recommendations Assessment, Development, and Evaluation (GRADE) framework.^16^ The certainty of evidence was downgraded based on RoB, inconsistency, indirectness, imprecision, and other relevant factors. The overall quality of evidence was classified as very low, low, moderate, or high. Summary of findings tables were generated using GRADEpro GDT (Guideline Development Tool).^17^

### Statistical analysis

We calculated the overall effect sizes by utilizing the mean changes and SD of several studies that looked into how yacon consumption affected weight, BMI, WC, and CRP. The data were displayed as bias-corrected weighted mean difference (WMD) and 95% CI to suitably depict the impact of yacon consumption on weight, BMI, WC, and CRP. We used the approved approach and relevant formulas to convert the results into the appropriate effect size for studies that did not disclose the mean (SD).^18^ By deducting each group’s postintervention measurements from its preintervention values, we calculated the mean difference between the weight, BMI, WC, and CRP of the intervention and control groups. The SD of weight, BMI, WC, and CRP in both intervention and control groups was calculated using the method “[√ (SD_1_^2^ + SD_2_)^2^- (2 × r × SD_1_ × SD_2_)]” under the assumption that the correlation coefficient (r) was 0.6.^18^^,^^19^ We used a random-effects model based on the Der Simonian and Laird approach to determine the overall impact size in order to account for the potential for variations among studies in the study.^20^^,^^21^ To evaluate the level of heterogeneity in a study, we employed Cochran's Q test and *I*^2^. Specifically, if the I-square is larger than 50% or the *P* value of Cochran's Q test is less than .05, we regard the study as having a considerable degree of heterogeneity.^22^ To give a more thorough interpretation of the results and, when significant, to pinpoint possible sources of heterogeneity, a subgroup analysis was carried out. To provide a thorough assessment, these subgroup analyses were predetermined, accounting for variables including participants’ mean age, sex, and study duration in weeks. The selection of these subgroup categories was informed by patterns and criteria established in previous research. To assess how each study affected the overall study findings, we conducted a sensitivity analysis. Potential publication bias was evaluated using funnel plots, Begg's rank correlation test, and Egger regression asymmetry.^23^ Additionally, the trim-and-fill method was used to evaluate the impact of missing studies on the pooled effect sizes, further examining potential publication bias. We performed sensitivity analysis using the Knapp-Hartung random-effects restricted (or residual, or reduced) maximum likelihood (REML) model and also calculated the 95% prediction interval for weight, BMI, and WC. When *P* values in this study were less than .05, we deemed statistical significance present. We utilized Stata version 14 software for analysis and Microsoft Excel for data extraction.

## Results

### Literature search

**Fig. 1:**
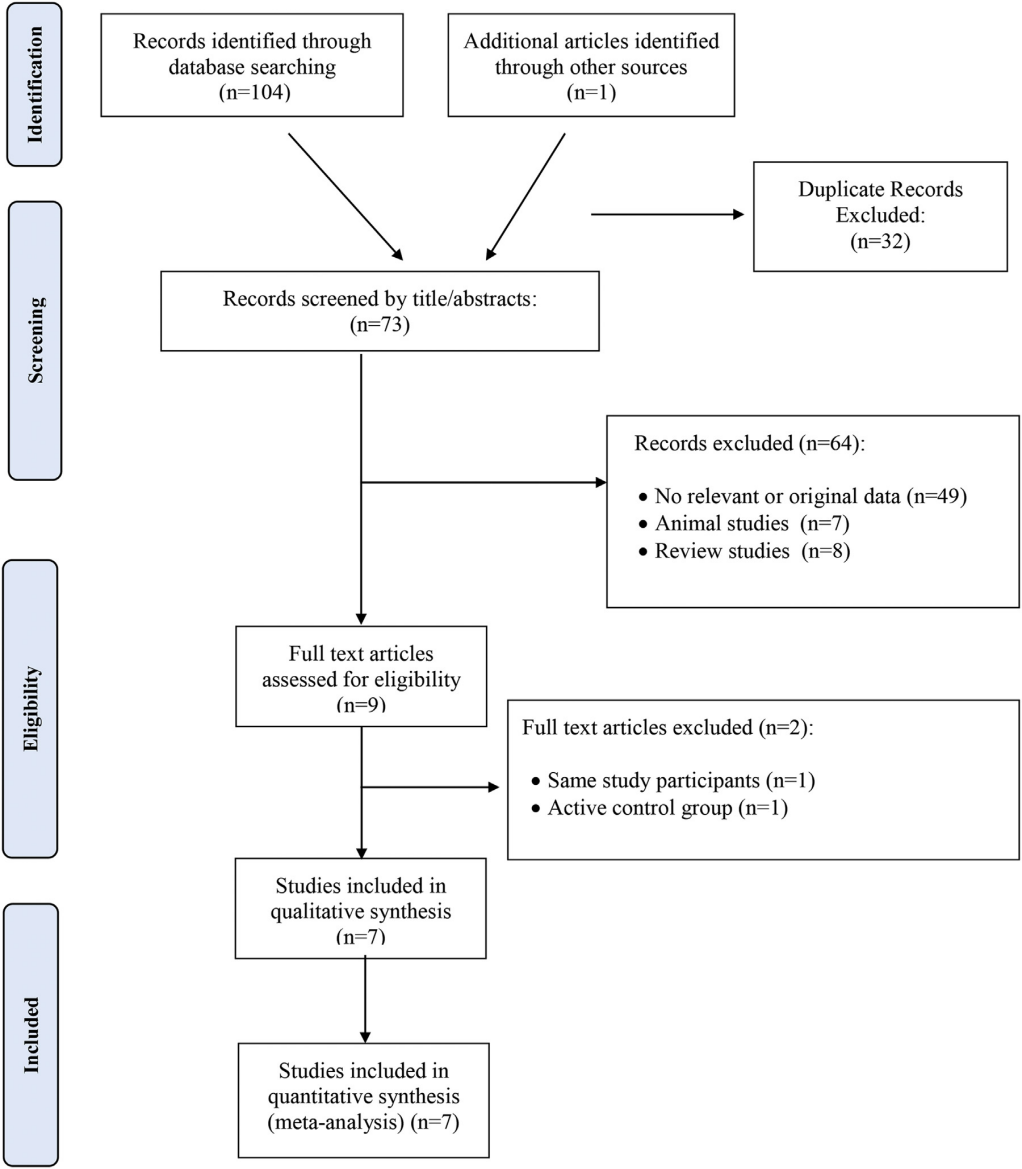
Flowchart of the number of studies identified and selected for the meta-analysis.

One hundred five publications were found in the first search using PubMed, Scopus, ISI Web of Sciences, and Google Scholar. A total of 73 papers were collected for title and abstract screening after 32 duplicates were eliminated. Following the review of titles and abstracts, 64 articles were removed (49 articles had no relevant or original data, 7 papers were animal studies, and 8 papers were review studies), and 9 primary studies were selected for a detailed full-text assessment. After full-text evaluation, one article had the same study participants,^24^ and 1 article had an active control group.^25^ Therefore, 7 papers were included in the meta-analysis. Figure 1 displays the study selection flow diagram.

### Study characteristics

Table 1^9^, ^10^, ^11^^,^^13^^,^^26^, ^27^, ^28^ lists the characteristics of 7 clinical trials that were included in our present systematic review. All articles were published between 2009 and 2024, with the majority conducted in Brazil,^9^, ^10^, ^11^^,^^13^^,^^27^ while others were conducted in Pakistan^26^ and Argentina.^28^ All papers were conducted using a parallel design. These studies were predominantly performed on healthy subjects,^9^, ^10^, ^11^^,^^13^^,^^27^ and others were executed on elderly subjects^26^ and slightly dyslipidemic premenopausal subjects.^28^ The majority of these studies included both male and female participants,^9^^,^^11^^,^^13^^,^^26^^,^^27^ while others focused exclusively on female subjects.^10^^,^^28^ In total, 246 participants were included with an age of 31.3 to 67.1 years and a mean baseline BMI of 25.1 to 33.7 kg/m^2^. Intervention durations ranged from 2 to 14 weeks. The studies encompassed various intervention and placebo groups, administering different doses of yacon in diverse forms (powder and syrup), along with varied placebo types. The outcomes measured across studies were heterogeneous, but primarily focused on weight, WC, BMI, and CRP levels.

**Table 1:** Demographic characteristics of the included studies.

Author (year)	Location	Study design	Health status	Sex	Sample size	Duration (wk)	Mean age (y)	Baseline BMI (kg/m2)	Intervention group	Comparator group	Outcome
Ashraf et al^26^ (2022)	Pakistan	RCT	Elderly Subjects	Both	20	8	NA	NA	20 g of yacon powder twice a day	Usual diet	Weight
Dionísio et al^27^ (2020)	Brazil	RCT	Healthy	Both	30	2	40.9	25.1	40 g yacon syrup	Placebo	Weight/BMI/WC/CRP
Genta et al^28^ (2009)	Argentina	RCT	Slightly dyslipidemic premenopausal	Female	55	14	40.5	33.5	0.14 g fructooligosaccharides/kg body weight/day	Placebo	Weight/BMI/WC
Machado et al^11^ (2021)	Brazil	RCT	Healthy	Both	26	6	31.3	30.4	25 g of yacon flour + breakfast drink (350 mL)	Breakfast drink (350 mL)	CRP
Scheid et al^9^ (2014)	Brazil	RCT	Healthy	Both	74	9	67.11	27.89	Freeze-dried powdered yacon	Placebo	CRP
de Melo Ribeiro et al^13^ (2021)	Brazil	RCT	Healthy	Both	26	6	31.3	30.4	25 g of yacon flour + breakfast drink (350 mL)	Breakfast drink (350 mL)	Weight/BMI/WC
Cabral et al^10^ (2024)	Brazil	RCT	Healthy	Female	15	4	38.3	33.74	12 mL of yacon syrup	12 mL of maltodextrin syrup (placebo)	Weight/BMI/WC

BMI = body mass index; CRP = C-reactive protein; NA = not available; RCT = randomized controlled trial; WC = waist circumference.

### Risk of bias assessment

The author’s complete evaluation of the article’s quality is given in Table 2.^9^, ^10^, ^11^^,^^13^^,^^26^, ^27^, ^28^ In conclusion, the included studies demonstrated a low risk of bias across most domains, including the randomization process, deviations from intended interventions, outcome measurement, and selective reporting. Only 2 studies^10^^,^^28^ were judged to have a high risk of bias due to missing outcome data. No study showed concerns in other domains.

**Table 2:** Risk of bias for randomized controlled trials, assessed according to the Revised Cochrane Risk of Bias Tool for randomized trials (RoB 2).

Publications	Randomization process	Deviations from the intended interventions	Missing out come data	Measurement of the outcome	Selection of the reported result
Ashraf et al^26^ (2022)	L	L	L	L	L
Dionísio et al^27^ (2020)	L	L	L	L	L
Genta et al^28^ (2009)	L	L	H	L	L
Machado et al^11^ (2021)	L	L	L	L	L
Scheid et al^9^ (2014)	L	L	L	L	L
de Melo Ribeiro et al^13^ (2021)	L	L	L	L	L
Cabral et al^10^ (2024)	L	L	H	L	L

H = high risk of bias; L = low risk of bias.

### GRADE assessment

The certainty of evidence was rated moderate for body weight and CRP, downgraded mainly due to inconsistency and imprecision, respectively. In contrast, evidence for BMI and WC was rated low owing to serious inconsistency (high heterogeneity) and imprecision, including a lack of significant effects. Overall, yacon appears to have more reliable evidence for weight reduction and CRP improvement, while findings for BMI and WC remain uncertain (Supplementary Materials).

### Findings from meta-analysis

**Fig. 2:**
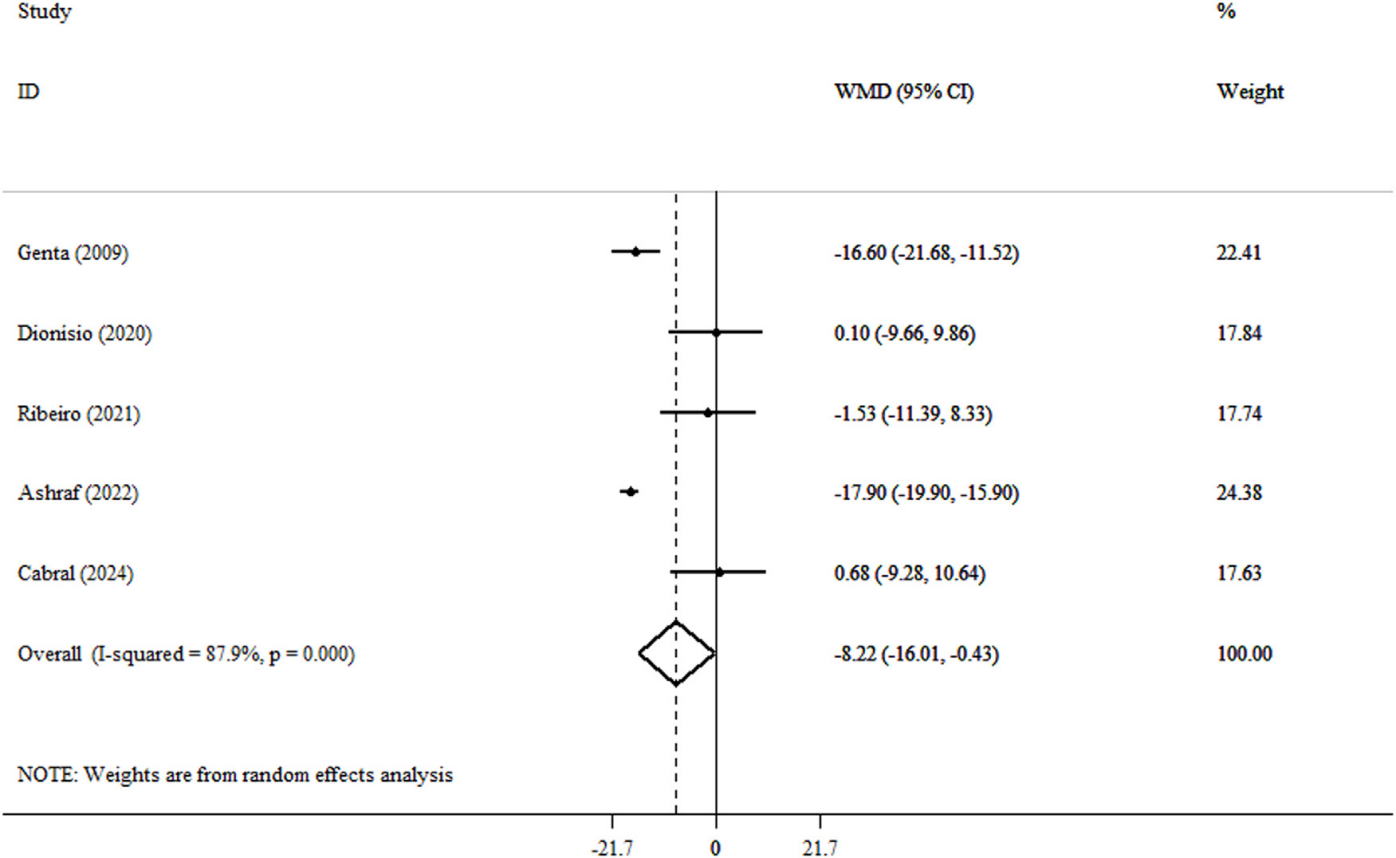
Forest plot detailing weighted mean difference (WMD) and 95% CIs for the effect of yacon on weight in participants (n = 146), with a median duration of 6 weeks of intervention.

**Fig. 3:**
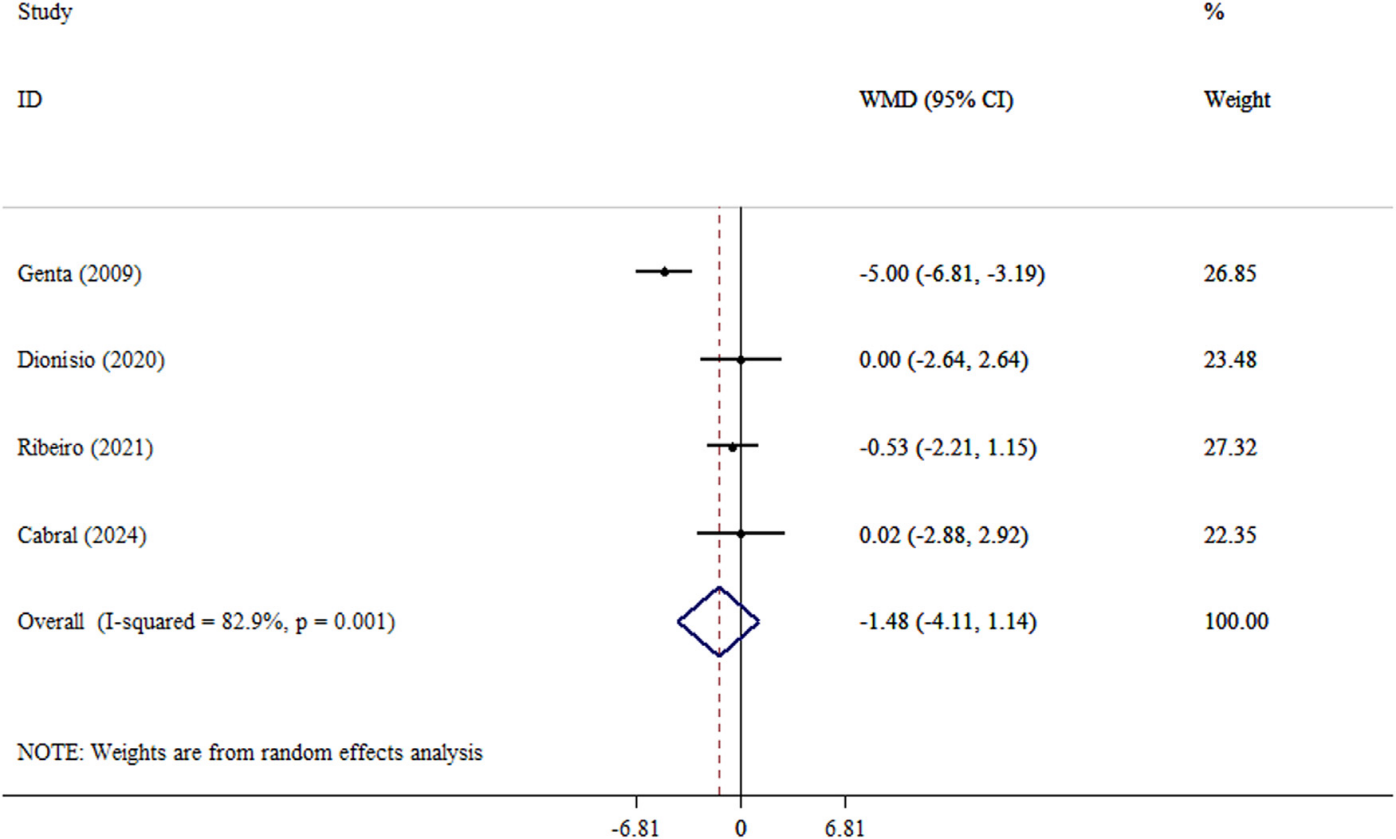
Forest plot detailing weighted mean difference (WMD) and 95% CIs for the effect of yacon on body mass index in participants (n = 126), with a median duration of 5 weeks of intervention.

**Fig. 4:**
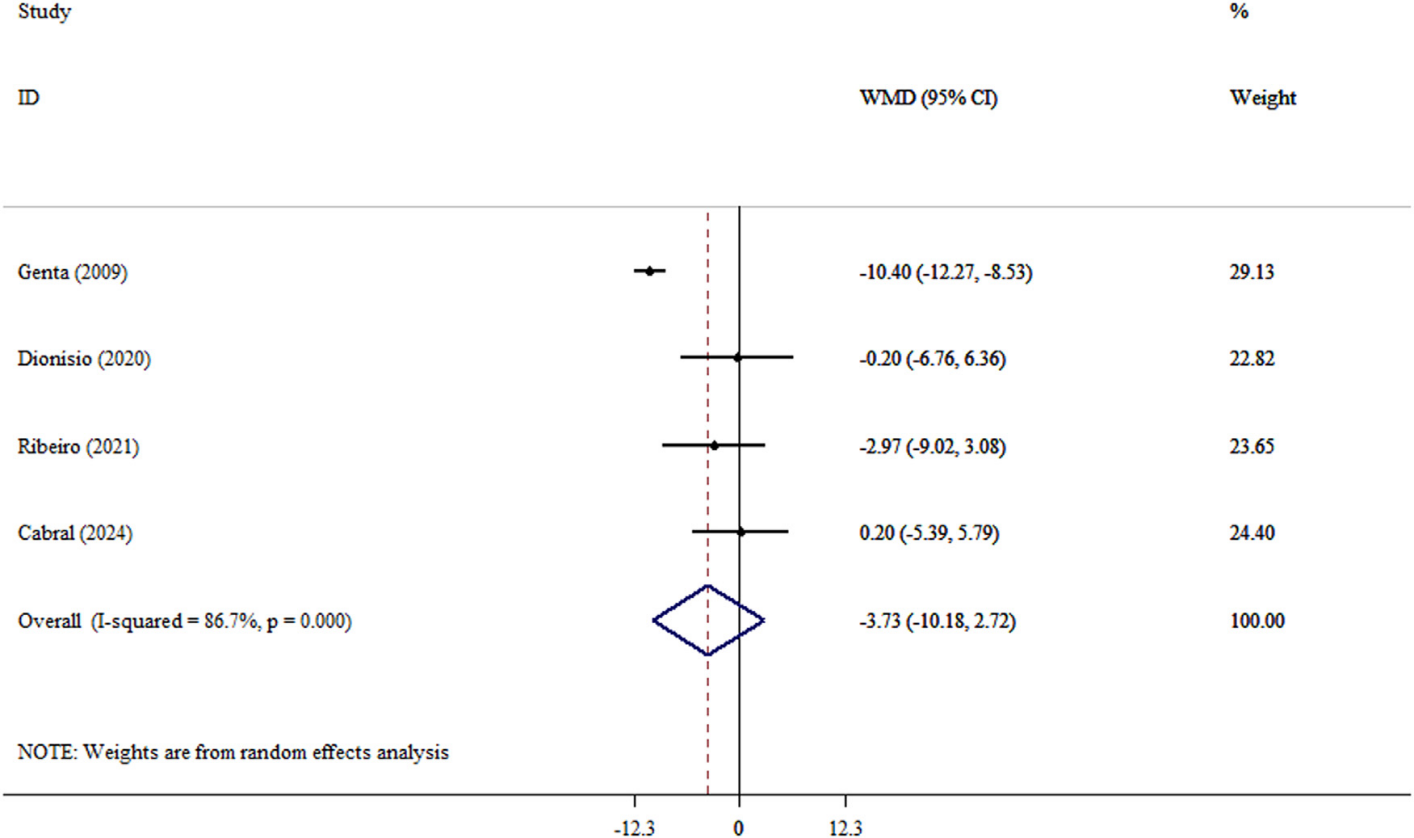
Forest plot detailing weighted mean difference (WMD) and 95% CIs for the effect of yacon on waist circumference in participants (n = 126), with a median duration of 5 weeks of intervention.

**Fig. 5:**
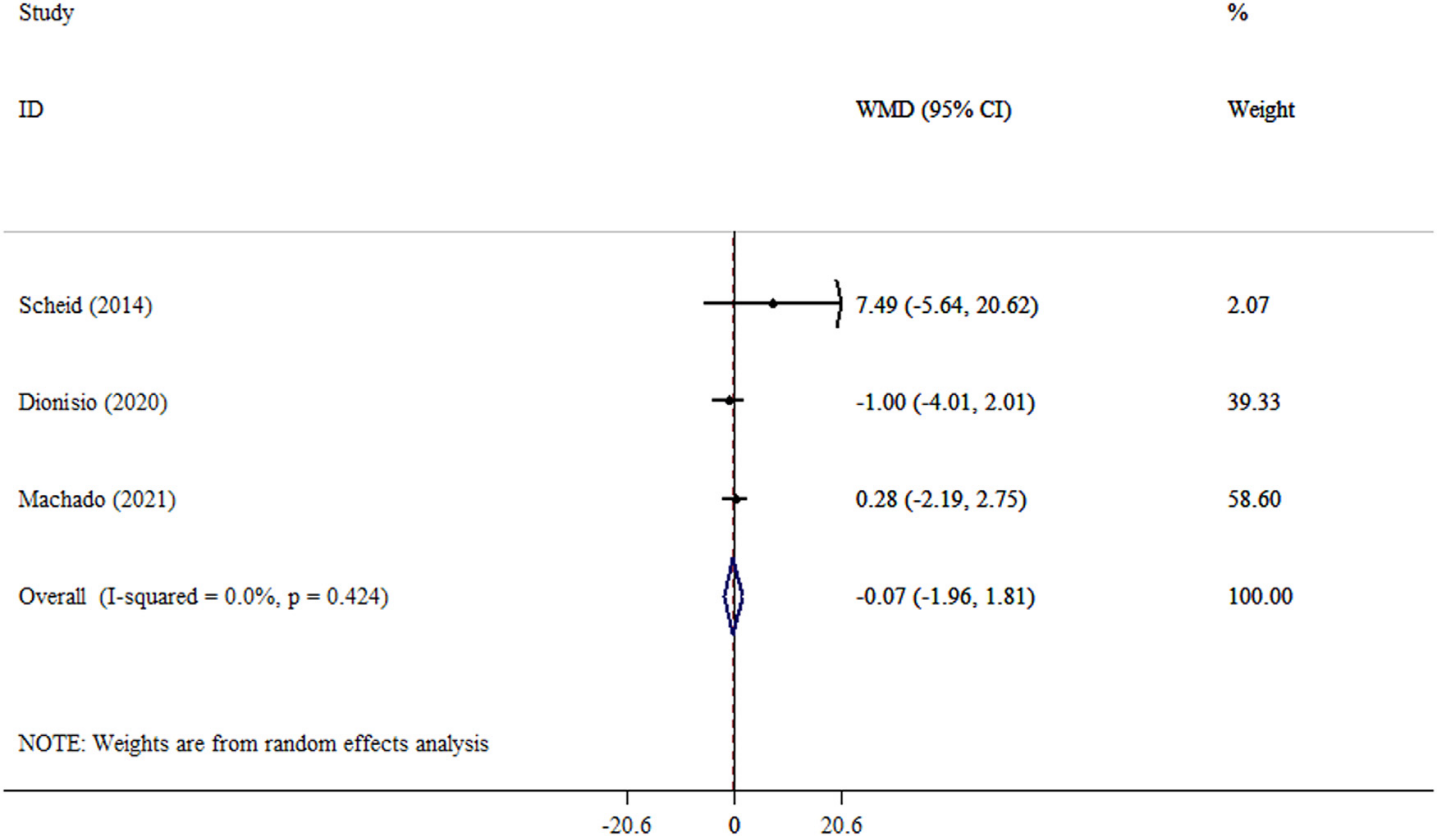
Forest plot detailing weighted mean difference (WMD) and 95% CIs for the effect of yacon on C-reactive protein in participants (n = 130), with a median duration of 6 weeks of intervention.

An analysis using the random effect model revealed a significant reduction in weight following yacon consumption (WMD, −8.22 kg; 95% CI, −16.01 to −0.43; *P* = .039; Cochran’s Q test = 33.0; tau^2^ = 63.7; *I*^2^ = 87.9%) (Figure 2 and Supplementary Materials). Significant heterogeneity is shown by *I*^2^ values greater than 85%, indicating that the pooled data should be regarded cautiously. The magnitude of weight loss observed with yacon consumption appears disproportionately large relative to the small sample sizes and short intervention durations of the included trials. Therefore, these findings should be interpreted cautiously. After analysis using the REML model, the results for weight were nonsignificant (MD, −8.02 kg; 95% prediction interval, −39.3, 23.3) (Supplementary Materials). However, no significant changes were observed in BMI (WMD, −1.48 kg/m^2^; 95% CI, −4.11 to 1.14; *P* = .268; Cochran’s Q test = 17.5; tau^2^ = 5.83; *I*^2^ = 82.9%) (Figure 3 and Supplementary Materials) or WC (WMD, −3.73 cm; 95% CI, −10.18 to 2.72; *P* = .257; Cochran’s Q test = 22.5; tau^2^ = 36.2; *I*^2^ = 86.7%) (Figure 4 and Supplementary Materials). After analysis using the REML model, the results for BMI and WC remained nonsignificant (MD, −1.49 kg/m^2^; 95% prediction interval, −5.44, 2.45), and (MD, −3.91 cm; 95% prediction interval, −12.1, 4.38) (Supplementary Materials). After excluding the outlier study,^28^ heterogeneity for the outcomes of BMI and WC was reduced. Also, after excluding outlier studies,^26^^,^^28^ heterogeneity for weight was reduced and became nonsignificant. Furthermore, yacon consumption did not significantly alter CRP levels, an established marker of systemic inflammation. This finding is clinically meaningful and should be interpreted in the context of the overall inflammatory profile (WMD, −0.07 mg/L; 95% CI, −1.96 to 1.81; *P* = .939; Cochran’s Q test = 1.72; tau^2^ < 0.001; *I*^2^ = 0.0) (Figure 5 and Supplementary Materials).

### Subgroup analysis

To address heterogeneity and present data more accurately, we performed subgroup analyses (Table 3) stratifying data by intervention duration, sex, and participant age. Results indicated that yacon consumption led to statistically significant reductions in weight, BMI, and WC, specifically among women aged 40 years and older, with weight reduction being more pronounced when the supplementation lasted longer than 8 weeks.

**Table 3:** Subgroup analysis of included randomized controlled trials in meta-analysis of the effect of yacon consumption on weight, BMI, and WC.

Group	No. of trials	No. of participants	WMD	95% CI	*P* value	*I**^2^* (%)	*P* heterogeneity	*P* for between subgroup heterogeneity
**Weight**								
Duration (wk)								<.001
<8	3	71	−0.25	−5.95 to 5.44	.93	0.0	.95	
≥8	2	75	−17.7	−19.5 to −15.8	<.001	0.0	.64	
Sex								<.001
Both	3	76	−0.25	−5.95 to 5.44	<.001	0.0	.95	
Female	2	70	−17.7	−19.5 to −15.8	<.001	0.0	.64	
Age (y)								<.001
<40	2	41	−0.44	−7.44 to 6.57	.90	0.0	.75	
≥40	3	105	−17.1	−18.9 to −15.2	<.001	84.1	.002	
**BMI**								
Sex								.003
Both	2	56	−0.38	−1.79 to 1.04	.60	0.0	.74	
Female	2	70	−3.60	−5.13 to −2.06	<.001	87.9	.004	
Age (y)								.005
<40	2	41	−0.39	−1.85 to 1.06	.59	0.0	.74	
≥40	2	85	−3.40	−4.89 to −1.91	<.001	89.4	.002	
**WC**								
Sex								.002
Both	2	56	−1.70	−6.14 to 2.75	.45	0.0	.54	
Female	2	70	−9.33	−11.1 to −7.56	<.001	91.9	<.001	
Age (y)								<.001
<40	2	41	−1.26	−5.37 to 2.85	.54	0.0	.45	
≥40	2	85	−9.63	−11.4 to −7.83	<.001	88.4	.003	

BMI = body mass index; WC = waist circumference; WMD = weighted mean difference.

### Sensitivity analyses

Influence analyses indicated that the findings for weight were not robust and sensitive to the exclusion of studies by Dionísio et al^27^ (2020), de Melo Ribeiro et al^13^, and Cabral et al^10^ (2024). Using correlation coefficients of 0.3 and 0.9 did not change the results (Supplementary Materials). Sensitivity analyses revealed that when small studies were eliminated, the pooled effect on body weight was not stable, indicating potential small-study bias.

### Publication bias

Visual inspection of the funnel plots suggested potential asymmetry for body weight and CRP (Supplementary Materials). However, formal statistical tests found no significant evidence of publication bias for BMI or CRP (Begg's test: *P* = .73 and *P* = .60; Egger's test: *P* = .57 and *P* = .43, respectively). Significant publication bias was detected for weight, with both Begg's (*P* = .05) and Egger's (*P* = .01) tests. The results for WC, however, were inconclusive, as Begg's test found no significant bias (*P* = .73) in contrast to Egger's test (*P* = .02). The Egger's and Begg's tests may have poor statistical power to identify publication bias due to the small number of included papers. To assess the potential influence of publication bias, a trim-and-fill analysis was performed. For weight, the analysis imputed 2 missing studies, resulting in a stronger yet still significant pooled estimate (−15.10 kg vs −8.22 kg), indicating a robust effect on weight reduction. For WC, the imputation of 2 studies rendered the previously nonsignificant estimate significant (−8.77, *P* = .004 vs −3.72, *P* = .25), suggesting that publication bias had obscured a true effect.

## Discussion

This meta-analysis sought to examine the impact of yacon consumption on anthropometric measurements and CRP levels. Significantly, it represents the first systematic review and meta-analysis to evaluate these effects specifically in an adult population. The analysis incorporated 7 studies, encompassing a total of 246 participants. The results indicate that yacon consumption is associated with meaningful reductions in weight. However, there were no significant alterations in BMI, WC, or CRP levels when compared to individuals who did not consume yacon.

According to the subgroup analysis, weight significantly decreased in individuals aged 40 years and older, in women, and among those who consumed yacon for more than 8 weeks. Additionally, yacon consumption is associated with reductions in BMI and WC, specifically in women and in individuals aged >40 years. However, no statistically significant differences were identified for other factors. However, because of the limited number of studies, the subgroup analysis is prespecified, indicating a need for further research with larger and more comprehensive samples to validate the findings.

A weight loss of 8.22 kg is clinically significant, as it surpasses the 5% to 10% body weight reduction threshold linked to meaningful health benefits, including improved metabolic markers and reduced chronic disease risk, additionally, it is greater than what is usually reported in prebiotic therapies (less than 3 kg over 8–12 weeks), indicating that care should be used when interpreting this effect as a possible physiological consequence. Despite being statistically significant, the reported 8 kg loss is unlikely to be physiologically achievable in 8 to 12 weeks and may instead be the result of confounding variables at the study level or methodological variability. Although the effect found in this meta-analysis approached statistical significance, its magnitude should be interpreted cautiously regarding practical clinical implications because clinically meaningful weight loss is commonly considered as a ≥5% drop in baseline body weight.^29^ Future RCTs should be preregistered since publication bias toward favorable results for functional foods cannot be ruled out. Due to the poor food monitoring in a number of the included trials, behavioral and placebo effects may potentially be involved. Yacon exhibits a comparable but inferior metabolic profile when compared to other prebiotics, such as inulin and resistant starch.^30^^,^^31^ However, achieving this weight loss through yacon consumption alone may be affected by several factors: individual variability, such as age, gender, baseline weight, metabolic rate, and genetics; dietary compliance, which relies on adherence to a balanced dietary strategy; and lifestyle factors, where regular physical activity plays a crucial role. Those who integrate yacon with lifestyle modifications are likely to experience more substantial and sustainable weight loss. Overall, this finding appears to be influenced by differences between studies rather than a direct effect of yacon. Furthermore, large prediction intervals restrict generalizability and show uncertainty. The absence of an anti-inflammatory effect of yacon on CRP levels can be justified by several factors, including the duration of the study. Most participants had baseline CRP levels within the normal range, which probably limited any discernible change. Shorter intervention periods may not be sufficient to observe significant changes in CRP, which is a marker of systemic inflammation. Additionally, individual variations in metabolism, baseline inflammatory status, and dietary factors could influence the results. It is also possible that the specific dosage of yacon consumed did not reach the threshold needed to elicit a measurable effect on CRP levels. C-reactive protein was selected as the primary inflammatory marker in this meta-analysis due to its established role as a sensitive and reliable indicator of systemic inflammation. However, focusing solely on CRP may limit the understanding of yacon’s broader anti-inflammatory potential. Future studies with longer durations, varied dosages, larger sample sizes, and calorie-matched control groups to better isolate may provide more insight into the potential anti-inflammatory effects of yacon on CRP and other inflammatory markers.

In line with our findings, human studies, overweight adults consuming a breakfast drink containing 25 g of yacon flour daily for 6 weeks showed reductions in body weight, body fat, WC, and sagittal abdominal diameter.^11^ Another study indicated that the consumption of yacon flour, combined with an energy-restricted diet, significantly decreased BMI and body weight.^13^ In contrast, a study by Cabral et al^10^ found that after 30 days of treatment with yacon, there was a significant reduction in body fat, but no significant changes were observed in body weight and BMI. Another study revealed that a 2-week consumption of yacon syrup did not promote significant changes in weight and BMI among healthy volunteers.^27^ One of the ways yacon root may promote weight loss is by reducing energy intake, as evidenced by our observation of decreased food consumption following yacon consumption. Dietary fiber reduces food intake by displacing nutrients, blocking nutrient absorption, and altering satiety hormones, leading to decreased appetite.^32^ Beneficial gut bacteria like *Lactobacillus* and *Bifidobacterium* use yacon-derived FOS as fermentable substrates to produce short-chain fatty acids (SCFAs) like butyrate, propionate, and acetate.^33^^,^^34^ By interacting with GPR41/43 receptors, these SCFAs improve inflammatory control, obesity, and energy balance.^35^ This mechanism, which has been mostly deduced from preclinical models, needs to be verified in human research.

Thus, the reduction of PPAR-γ, C/EBPα, and aP2 expression in the visceral adipose tissue due to yacon consumption may inhibit adipogenesis, improve insulin sensitivity, and decrease blood lipid levels.^36^

Yacon exhibits anti-inflammatory properties through several mechanisms. It is rich in bioactive compounds such as polyphenols and flavonoids, which provide antioxidant effects that help neutralize free radicals and reduce oxidative stress linked to inflammation.^37^ Additionally, the prebiotic FOS in yacon promotes the growth of beneficial gut bacteria, contributing to a balanced microbiome that modulates immune responses and lowers the production of pro-inflammatory cytokines.^38^ Yacon consumption can reduce inflammation by lowering cytokines like IL-6 and TNF-α, inhibiting the NF-κB pathway, improving glucose metabolism and insulin sensitivity, and producing anti-inflammatory SCFAs through gut fermentation.^33^

### Biological plausibility

Yacon has been shown to have anti-inflammatory and prebiotic properties through improved immunological regulation, gut microbial diversity, and SCFA synthesis in animal models. However, because species-specific variations in metabolism and dose make translational relevance to humans unknown, these results should be regarded with caution.^7^^,^^39^^,^^40^ This meta-analysis has several advantages, particularly as it represents the first comprehensive study to investigate these effects. This meta-analysis enhances the understanding of yacon as a dietary intervention for weight management and inflammation. Although existing meta-analyses have often concentrated on well-established prebiotics such as inulin or psyllium, which primarily emphasize their effects on gut health and glycemic control, this review specifically highlights the unique properties of yacon, including its rich FOS content and low energy density. Furthermore, this meta-analysis uniquely synthesizes findings across diverse formulations of yacon, such as syrup and flour, and evaluates their differential impacts, thereby providing a more comprehensive understanding of yacon’s potential in clinical practice

It includes all relevant studies without language restrictions, ensuring a more inclusive data set. This approach allows for a broader interpretation of the findings and reduces potential biases associated with language limitations. Additionally, the meta-analysis employs rigorous methodologies, enhancing the reliability and validity of the results. By synthesizing data from various studies, it provides a thorough understanding of the effects under investigation and sets a foundation for future research in the field. However, several limitations must be considered when interpreting the findings of this meta-analysis. Many of the trials included in the review exhibited a moderate to high risk of bias, which impacts the reliability of the reported outcomes and necessitates cautious interpretation. The high heterogeneity (*I*^2^ of 87%–88%) across studies suggests significant variability in results. The studies varied significantly in terms of participant age and health profiles (healthy vs metabolic disorders), the dosages administered, the duration of interventions, yacon formulations (syrup vs powder), and the control conditions used for comparison. These factors may influence the effectiveness of yacon and should be considered in future research and the interpretation of findings. Moreover, dietary context, such as energy restriction versus ad libitum eating, influences the effects of yacon consumption. Energy restriction may enhance yacon’s metabolic benefits, while unrestricted intake could lessen them, contributing to variability in study results. Recognizing this factor is important for understanding how yacon’s efficacy depends on dietary conditions. These substantial differences among the trials made it inappropriate to statistically combine the results in the meta-analysis. Subgroup analyses offered some intriguing insights (such as larger impacts in women and people aged >40 years), but these results should be viewed cautiously. The observed connections might partially be the result of chance findings rather than actual impacts because many subgroup analyses raise the possibility of type I error inflation. We recognize the methodological restriction that this meta-analysis was not preregistered in PROSPERO. To validate the potential health benefits of yacon identified in this review, there is a clear need for higher-quality studies with larger sample sizes. Although yacon is marketed for weight loss and digestive health, it is crucial to distinguish evidence-based insights from consumer-driven claims. Many marketing assertions lack rigorous scientific backing, necessitating further validation through well-designed studies. Confirming yacon’s effectiveness could influence food policies and clinical diets by encouraging its use as a natural option to improve metabolic health, impacting public health and personalized nutrition. In light of these constraints, future studies should be preregistered in PROSPERO to improve methodological transparency, focus on standardized yacon preparations with measured FOS levels, include calorie-matched control groups, evaluate inflammatory markers like TNF-α and IL-6, and incorporate gut microbiota analyses. Results may be skewed by behavioral factors, such as food changes or heightened health confound during supplementation. To reduce this bias, dietary monitoring should be a part of future RCTs. Future research should also accurately measure FOS concentration, incorporate multi-omics techniques (microbiome, metabolomics, transcriptomics) to clarify processes, and involve a variety of populations outside of Brazilian cohorts. Research is also necessary to determine the long-term safety of regular yacon consumption.

### Conclusion

According to a comprehensive review and meta-analysis, yacon consumption in adults is linked to significant weight reductions, although it does not affect BMI, WC, or CRP levels. Nevertheless, there is evidence suggesting that long-term yacon consumption may lead to improved weight over an 8-week period, particularly in women and individuals aged >40 years. Additionally, yacon may positively influence WC and BMI in these groups. These findings suggest that while immediate effects on CRP and other anthropometric measures may not be evident, prolonged use of yacon could provide potential benefits. Current findings should be regarded cautiously due to the substantial heterogeneity of evidence and moderate certainty. Rather than acting as a stand-alone strategy, Yacon may be used as a supportive dietary supplement for weight management. From the standpoint of public health, yacon is a functional food that shows promise but is still under inquiry and deserves more investigation in extensive, standardized clinical trials.

## Funding

Not applicable.

## CRediT Authorship Contribution Statement

**Fariborz Porbafrani**: Data curation, Formal analysis, Methodology, Writing – original draft. **Mohammad Samadi**: Methodology, Writing – original draft. **Karim Parastouei**: Supervision, Writing – review & editing.

## Human Ethics and Consent to Participate Declarations

All included RCTs had ethical approval and participant consent.

## Data Availability

All data extraction sheets, sensitivity analyses, and related materials are available in the supplementary materials associated with this article. The data used to support the findings of this study are available from the corresponding author upon request.

## Declaration of competing interest

The authors declare that they have no known competing financial interests or personal relationships that could have appeared to influence the work reported in this paper.

## Appendix Supplementary materials

Image, application 1
