# Supplementary material for: The Effects of Yacon Consumption on Body Weight and C-reactive Protein: A Systematic Review and Meta-analysis of Randomized Controlled Trials
Source: Curr Ther Res Clin Exp. 2025 Dec 17;104:100817. doi: 10.1016/j.curtheres.2025.100817 (PMC12890843; doi:10.1016/j.curtheres.2025.100817)
Supplement: Supplementary file 1 [file mmc1.docx]

**Supplementary Table 1.** PRISMA Checklist.

| **Section/topic** | | **#** | | | **Checklist item** | **Reported on page #** |
| --- | --- | --- | --- | --- | --- | --- |
| **TITLE** | | | | | |  |
| Title | | 1 | | | Identify the report as a systematic review, meta-analysis, or both. | Page:1 |
| **ABSTRACT** | | | | | |  |
| Structured summary | | 2 | | | Provide a structured summary including, as applicable: background; objectives; data sources; study eligibility criteria, participants, and interventions; study appraisal and synthesis methods; results; limitations; conclusions and implications of key findings; systematic review registration number. | Page: 2 |
| **INTRODUCTION** | | | | | |  |
| Rationale | | 3 | | | Describe the rationale for the review in the context of what is already known. | Page: 3 |
| Objectives | | 4 | | | Provide an explicit statement of questions being addressed with reference to participants, interventions, comparisons, outcomes, and study design (PICOS). | Page: 4 |
| **METHODS** | | | | | |  |
| Protocol and registration | 5 | | Indicate if a review protocol exists, if and where it can be accessed (e.g., Web address), and, if available, provide registration information including registration number. | | | Page: 4 |
| Eligibility criteria | 6 | | Specify study characteristics (e.g., PICOS, length of follow-up) and report characteristics (e.g., years considered, language, publication status) used as criteria for eligibility, giving rationale. | | | Page:5 |
| Information sources | 7 | | Describe all information sources (e.g., databases with dates of coverage, contact with study authors to identify additional studies) in the search and date last searched. | | | Page: 4 |
| Search | 8 | | Present full electronic search strategy for at least one database, including any limits used, such that it could be repeated. | | | Page: 4 |
| Study selection | 9 | | State the process for selecting studies (i.e., screening, eligibility, included in systematic review, and, if applicable, included in the meta-analysis). | | | Page: 5 |
| Data collection process | 10 | | Describe method of data extraction from reports (e.g., piloted forms, independently, in duplicate) and any processes for obtaining and confirming data from investigators. | | | Page: 6, 8 |
| Data items | 11 | | List and define all variables for which data were sought (e.g., PICOS, funding sources) and any assumptions and simplifications made. | | | Page 4,5 |
| Risk of bias in individual studies | 12 | | Describe methods used for assessing risk of bias of individual studies (including specification of whether this was done at the study or outcome level), and how this information is to be used in any data synthesis. | | | Page 6 |
| Summary measures | 13 | | State the principal summary measures (e.g., risk ratio, difference in means). | | | Page 7 |
| Synthesis of results | 14 | | Describe the methods of handling data and combining results of studies, if done, including measures of consistency (e.g., I^2^) for each meta-analysis. | | | Page 7 |
| Risk of bias across studies | 15 | | | Specify any assessment of risk of bias that may affect the cumulative evidence (e.g., publication bias, selective reporting within studies). | | Page 6 |
| Additional analyses | 16 | | | Describe methods of additional analyses (e.g., sensitivity or subgroup analyses, meta-regression), if done, indicating which were pre-specified. | | Page 7-8 |
| **RESULTS** | | | | | |  |
| Study selection | 17 | | | Give numbers of studies screened, assessed for eligibility, and included in the review, with reasons for exclusions at each stage, ideally with a flow diagram. | | Page 8-9 |
| Study characteristics | 18 | | | For each study, present characteristics for which data were extracted (e.g., study size, PICOS, follow-up period) and provide the citations. | | Page 8-9 |
| Risk of bias within studies | 19 | | | Present data on risk of bias of each study and, if available, any outcome level assessment (see item 12). | | Page 9 |
| Results of individual studies | 20 | | | For all outcomes considered (benefits or harms), present, for each study: (a) simple summary data for each intervention group (b) effect estimates and confidence intervals, ideally with a forest plot. | | Page 9-10 |
| Synthesis of results | 21 | | | Present results of each meta-analysis done, including confidence intervals and measures of consistency. | | Page 9-10 |
| Risk of bias across studies | 22 | | | Present results of any assessment of risk of bias across studies (see Item 15). | | Page 9 |
| Additional analysis | 23 | | | Give results of additional analyses, if done (e.g., sensitivity or subgroup analyses, meta-regression [see Item 16]). | | Page 9-10 |
| **DISCUSSION** | | | | | |  |
| Summary of evidence | 24 | | | Summarize the main findings including the strength of evidence for each main outcome; consider their relevance to key groups (e.g., healthcare providers, users, and policy makers). | | Page 12 |
| Limitations | 25 | | | Discuss limitations at study and outcome level (e.g., risk of bias), and at review-level (e.g., incomplete retrieval of identified research, reporting bias). | | Page 17 |
| Conclusions | 26 | | | Provide a general interpretation of the results in the context of other evidence, and implications for future research. | | Page 17-18. |
| **FUNDING** | | | | | |  |
| Funding | 27 | | | Describe sources of funding for the systematic review and other support (e.g., supply of data); role of funders for the systematic review. | | Page 18 |

**Supplementary Table 2.** Search syntax.

| **Search syntax** | |
| --- | --- |
| (Yacon[tiab] OR "Smallanthus sonchifolius"[tiab]) AND (intervention[tiab] OR RCT[tiab] OR randomized[tiab] OR random[tiab] OR Randomly[tiab] OR Placebo[tiab] OR Assignment[tiab] OR trial[tiab] OR trials[tiab] OR randomised[tiab] OR "Methods"[Mesh] OR Cross-Over[tiab] OR "Double-Blind"[tiab] OR "Randomized Controlled Trial"[Publication Type] OR "Controlled Clinical Trial"[Publication Type] OR "Placebos"[Mesh] OR "Placebo Effect"[Mesh] OR "Clinical Trial"[Publication Type] OR "Clinical Trials as Topic"[Mesh] OR "Cross-Over Studies"[Mesh] OR "Double-Blind Method"[Mesh]) | **Pubmed** |
| ( TITLE-ABS-KEY ( Yacon ) OR TITLE-ABS-KEY ( "Smallanthus sonchifolius" ) AND TITLE-ABS-KEY ( intervention ) OR TITLE-ABS-KEY ( "controlled trial" ) OR TITLE-ABS-KEY ( randomized ) OR TITLE-ABS-KEY ( random ) OR TITLE-ABS-KEY ( randomly ) OR TITLE-ABS-KEY ( placebo ) OR TITLE-ABS-KEY ( assignment ) OR TITLE-ABS-KEY ( "clinical trial" ) OR TITLE-ABS-KEY ( trial ) OR TITLE-ABS-KEY ( randomised ) ) AND ( LIMIT-TO ( DOCTYPE , "ar" ) ) AND ( LIMIT-TO ( LANGUAGE , "English" ) ) AND ( LIMIT-TO ( SRCTYPE , "j" ) ) | **Scopus** |
| Yacon OR "Smallanthus sonchifolius" (All Fields) and intervention OR RCT OR randomized OR random OR Randomly OR Placebo OR Assignment OR trial OR trials OR randomized OR Cross-Over OR "Double-Blind" (All Fields) | **Web of Science** |
| (Yacon OR "Smallanthus sonchifolius"):ti,ab,kw AND (intervention OR RCT OR randomized OR random OR Randomly OR Placebo OR Assignment OR trial OR trials OR randomized OR Cross-Over OR "Double-Blind"):ti,ab,kw | **Cochrane Library** |

| **Supplementary Table 3.** GRADE profile of yacon consumption on anthropometric measures and C-reactive protein. | | | | | | |
| --- | --- | --- | --- | --- | --- | --- |
| **Outcomes** | **Risk of bias** | **Inconsistency** | **Indirectness** | **Imprecision** | **Publication Bias** | **Quality of evidence** |
| **Weight (kg)** | No serious limitations | ^a^Serious limitations | No Serious Limitations | No Serious Limitations | No serious limitations | ⊕⊕⊕◯ Moderate |
| **BMI (kg/m^2)^** | No serious limitations | ^b^Serious limitations | No Serious Limitations | ^d^Serious Limitations | No serious limitations | ⊕⊕◯◯ LOW |
| **WC (cm)** | No serious limitations | ^c^Serious limitations | No Serious Limitations | Serious Limitations | No serious limitations | ⊕⊕◯◯ LOW |
| **CRP (mg/l)** | No serious limitations | No serious limitations | No Serious Limitations | Serious Limitations | No serious limitations | ⊕⊕⊕◯ Moderate |
| ^a^ Serious Inconsistency since I^2^= 87.9%, Downgraded.  ^a^ Serious Inconsistency since I^2^= 82.9%, Downgraded.  ^a^ Serious Inconsistency since I^2^= 86.7%, Downgraded.  ^d^ There is no significant effect of yacon intake | | | | | | |

| **Supplementary Table 4**. Weighted mean difference and 95% confidence intervals (CIs) for the effect of yacon consumption on anthropometric and CRP. | | |
| --- | --- | --- |
| **Group** | **WMD (95% CI)** | ***I^2^* (%)** |
| **Correlation coefficient (r) assumed to be 0.3** | | |
| ***Weight (kg)*** | -9.07 (-16.9, -1.17) | 79.5 |
| ***BMI (kg/m^2)^*** | -1.56 (-4.21, 1.09) | 70.9 |
| ***WC (cm)*** | -4.09 (-10.5, 2.45) | 76.9 |
| ***CRP (mg/l)*** | 0.02 (-2.45, 2.49) | 0.00 |
| **Correlation coefficient (r) assumed to be 0.9** | | |
| ***Weight (kg)*** | -7.31 (-15.1, 0.53) | 96.5 |
| ***BMI (kg/m^2)^*** | -1.39 (-3.87, 1.07) | 94.8 |
| ***WC (cm)*** | -3.43 (-9.79, 2.91) | 96.5 |
| ***CRP (mg/l)*** | -0.19 (-1.57, 1.18) | 32.6 |
| Abbreviations: **BMI,** Body mass index; **WC,** Waist circumference; **CRP,** C-reactive protein; **WMD**, Weight mean difference; | | |

**Supplementary Table 5**. Summary of baseline and final data (mean ± SD) and change scores for included studies (correlation coefficient (r) assumed to be 0.6).

| **First author** | **Baseline** | | **Final** | | **Baseline** | | **Final** | | **Baseline change** | | **Final change** | |
| --- | --- | --- | --- | --- | --- | --- | --- | --- | --- | --- | --- | --- |
|  | **In_Mean** | **In_SD** | **In_Mean** | **In_SD** | **Pl_Mean** | **Pl_SD** | **Pl_Mean** | **Pl_SD** | **In_Mean** | **In_SD** | **Pl_Mean** | **Pl_SD** |
| ***Weight (kg)*** | | | | | | | |  | |  | | |
| Ashraf et al. | 85.6 | 2.8 | 73.8 | 0.3 | 75.4 | 2.32 | 81.5 | 1.37 | -11.8 | 2.63 | 6.1 | 1.85 |
| Dionísio et al. | 69.1 | 14 | 69.2 | 14 | 71.2 | 16.4 | 71.2 | 16.4 | 0.1 | 12.5 | 0 | 14.6 |
| Genta et al. | 91.2 | 8.4 | 76.2 | 6.1 | 90.7 | 10.3 | 92.3 | 10.1 | -15 | 6.80 | 1.6 | 9.12 |
| Ribeiro et al. | 88.52 | 15.21 | 85.95 | 14.67 | 84.93 | 13.83 | 83.89 | 13.54 | -2.57 | 13.3 | -1.04 | 12.2 |
| Cabral et al. | 87.16 | 10.07 | 86.44 | 10.63 | 78.46 | 10.42 | 77.06 | 10.33 | -0.72 | 9.27 | -1.4 | 9.28 |
| ***BMI*** ***(kg/m^2)^*** | | | | | | | | | | | | |
| Dionísio et al. | 25.3 | 3.3 | 25.3 | 3.3 | 25 | 4.8 | 25 | 4.8 | 0 | 2.95 | 0 | 4.29 |
| Genta et al. | 34 | 2 | 28 | 3 | 33 | 3 | 32 | 4 | -6 | 2.40 | -1 | 3.25 |
| Ribeiro et al. | 30.8 | 2.85 | 29.91 | 2.82 | 30.08 | 2.04 | 29.72 | 1.92 | -0.89 | 2.53 | -0.36 | 1.77 |
| Cabral et al. | 35.34 | 3.66 | 35.04 | 3.92 | 32.14 | 2.64 | 31.82 | 2.45 | -0.3 | 3.39 | -0.32 | 2.28 |
| ***WC (cm)*** | | | | | | | | | | | | |
| Dionísio et al. | 82.9 | 8.2 | 82.3 | 8 | 82.9 | 11.7 | 82.5 | 12.3 | -0.6 | 7.24 | -0.4 | 10.7 |
| Genta et al. | 105.1 | 5 | 95.2 | 4.8 | 101.4 | 3.1 | 101.9 | 2.4 | -9.9 | 4.38 | 0.5 | 2.53 |
| Ribeiro et al. | 102.62 | 9.38 | 88.13 | 8.45 | 100.18 | 8.19 | 88.66 | 9.02 | -14.49 | 8.01 | -11.52 | 7.73 |
| Cabral et al. | 94.89 | 6.61 | 93.89 | 7.43 | 89.38 | 4.83 | 88.18 | 5.3 | -1 | 6.32 | -1.2 | 4.54 |
| ***CRP (mg/l)*** | | | | | | | | | | | | |
| Dionísio et al. | 2 | 1 | 2 | 1 | 4 | 6 | 5 | 7 | 0 | 0.89 | 1 | 5.88 |
| Machado et al. | 4.89 | 3.85 | 5.23 | 4.21 | 2.52 | 3.02 | 2.58 | 3.1 | 0.34 | 3.61 | 0.06 | 2.73 |
| Scheid et al. | 3.66 | 5.03 | 3.78 | 4.46 | 10.36 | 42.39 | 2.99 | 3.26 | 0.12 | 4.27 | -7.37 | 40.5 |
| Abbreviations: **In,** Intervention; **Pl,** Placebo; **SD**, Standard deviation; **BMI**, Body mass index; **WC,** Waist circumference; **CRP,** C-reactive protein; | | | | | | | | | | | | |

**Supplementary Table 6**. List of rules for data estimation and conversion.

| **Obtaining SD from SE** |
| --- |
| SD= Se.$\surd n$ |
| **Obtaining SD change from SD and r** |
| SD change = [√ (SD_1_^2^ + SD_2_)^2^- (2 × r × SD_1_ × SD_2_)] |


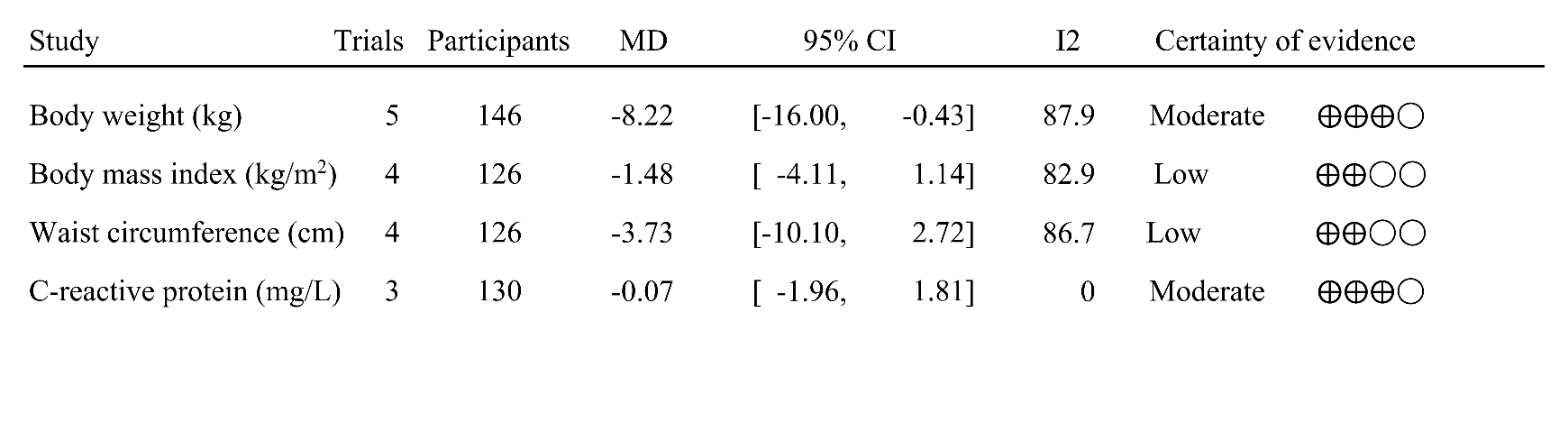


**Supplementary Figure 1**. Summary results of the effect of yacon consumption on anthropometric measures and CRP.

**Supplementary Figure 2.** Forest plot detailing weighted mean difference and 95% confidence intervals (CIs) for the effect of yacon on weight.

**Supplementary Figure 3.** Forest plot detailing weighted mean difference and 95% confidence intervals (CIs) for the effect of yacon on BMI.

**Supplementary Figure 4.** Forest plot detailing weighted mean difference and 95% confidence intervals (CIs) for the effect of yacon on WC.


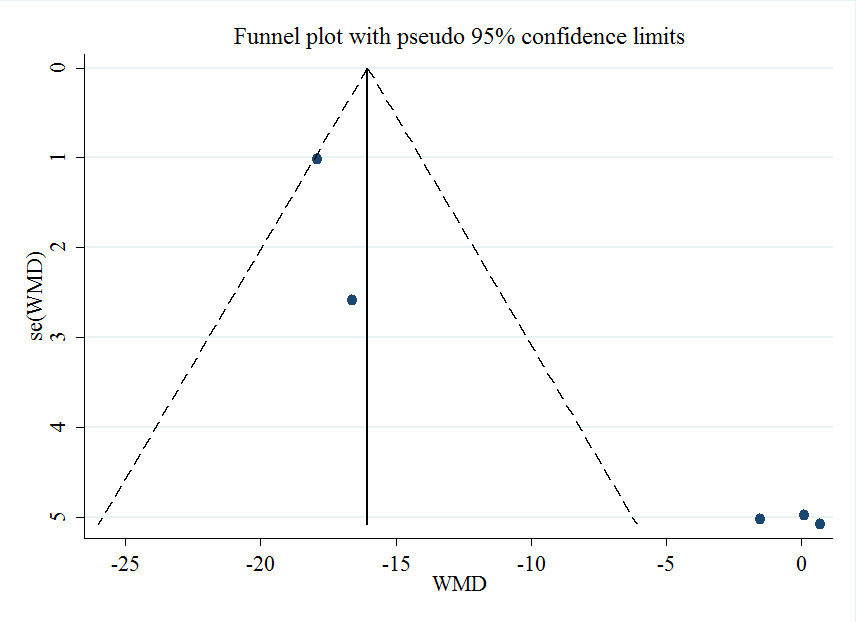


**Supplementary Figure 5.** Funnel plot displaying publication bias in the studies reporting the impact of yacon on weight. Egger's test (p = 0.01).


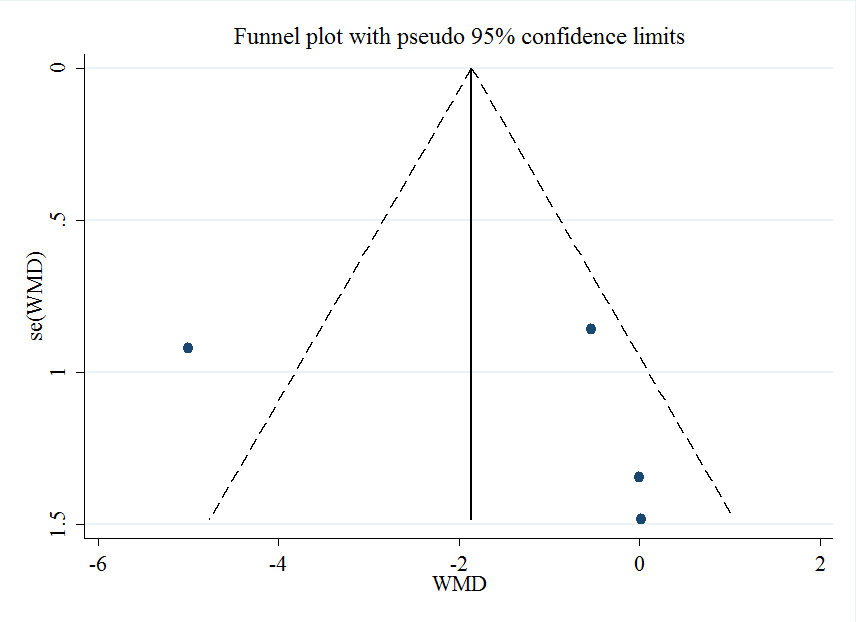


**Supplementary Figure 6.** Funnel plot displaying publication bias in the studies reporting the impact of yacon on BMI. Egger's test (p=0.57).


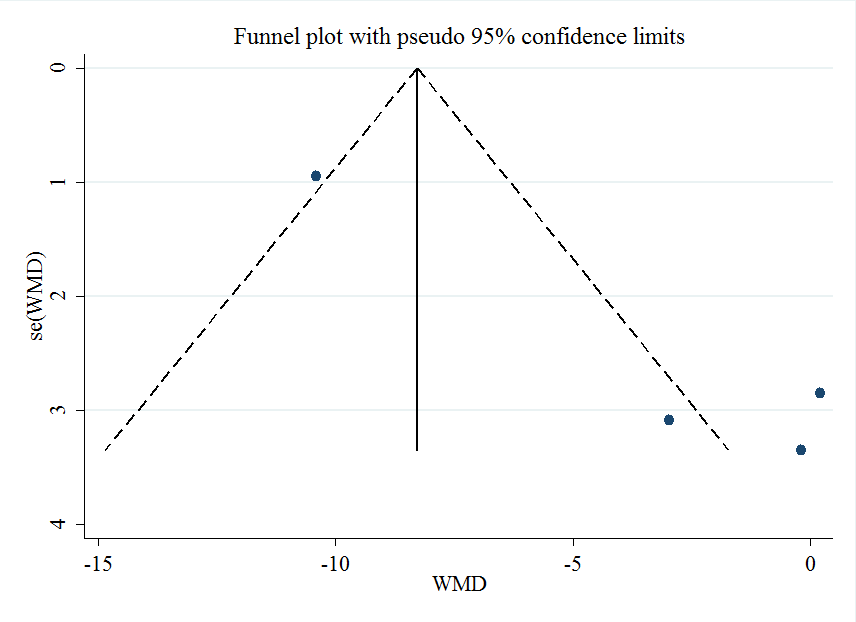


**Supplementary Figure 7.** Funnel plot displaying publication bias in the studies reporting the impact of yacon on WC. Egger's test (p = 0.02).


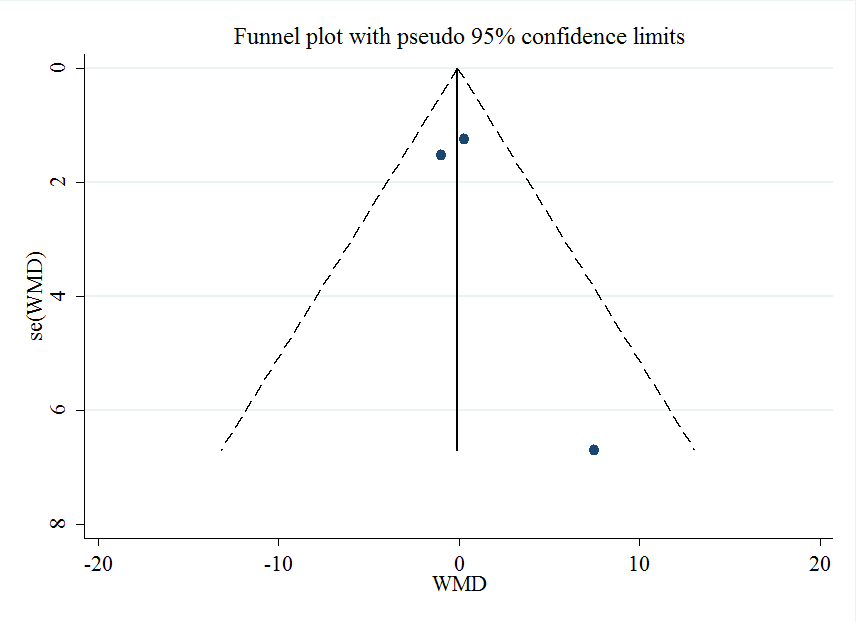


**Supplementary Figure 8.** Funnel plot displaying publication bias in the studies reporting the impact of yacon on CRP. Egger's test (p= 0.43).

**
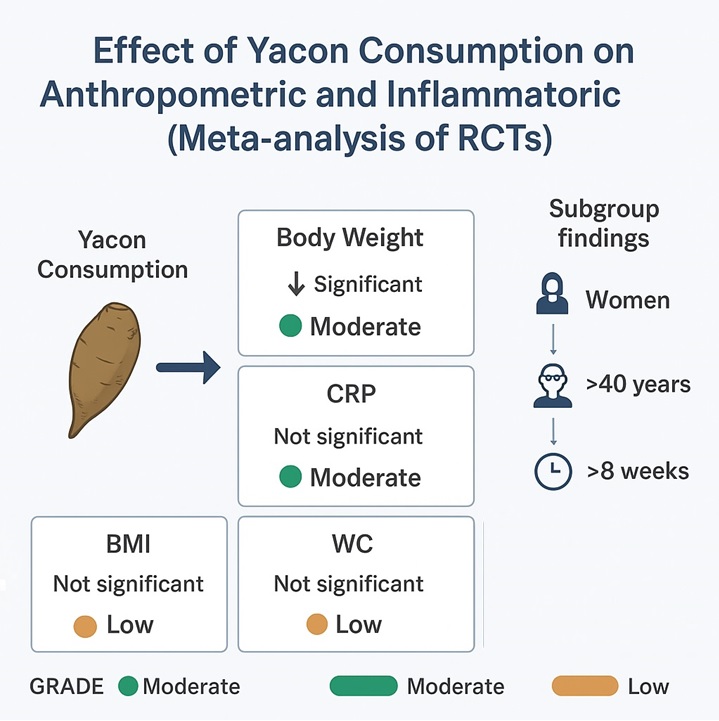
**

**Supplementary Figure 9.** Graphical Abstract.

**Supplementary Figure 10.** Sensitivity analysis for the effect of yacon on weight.

**Supplementary Figure 11.** Sensitivity analysis for the effect of yacon on BMI.

**Supplementary Figure 12.** Sensitivity analysis for the effect of yacon on WC.

**Supplementary Figure 13.** Sensitivity analysis for the effect of yacon on CRP.
